# Comparative efficacy of selenate and selenium nanoparticles for improving growth, productivity, fruit quality, and postharvest longevity through modifying nutrition, metabolism, and gene expression in tomato; potential benefits and risk assessment

**DOI:** 10.1371/journal.pone.0244207

**Published:** 2020-12-18

**Authors:** Maryam Neysanian, Alireza Iranbakhsh, Rahim Ahmadvand, Zahra Oraghi Ardebili, Mostafa Ebadi

**Affiliations:** 1 Department of Biology, Science and Research Branch, Islamic Azad University, Tehran, Iran; 2 Department of Seed and Plant Research Improvement Institute, Karaj, Iran; 3 Department of Biology, Garmsar Branch, Islamic Azad University, Garmsar, Iran; 4 Department of Biology, Damghan Branch, Islamic Azad University, Damghan, Iran; University of Pittsburgh, UNITED STATES

## Abstract

This study attempted to address molecular, developmental, and physiological responses of tomato plants to foliar applications of selenium nanoparticles (nSe) at 0, 3, and 10 mgl^-1^ or corresponding doses of sodium selenate (BSe). The BSe/nSe treatment at 3 mgl^-1^ increased shoot and root biomass, while at 10 mgl^-1^ moderately reduced biomass accumulation. Foliar application of BSe/nSe, especially the latter, at the lower dose enhanced fruit production, and postharvest longevity, while at the higher dose induced moderate toxicity and restricted fruit production. In leaves, the BSe/nSe treatments transcriptionally upregulated miR172 (mean = 3.5-folds). The Se treatments stimulated the expression of the *bZIP* transcription factor (mean = 9.7-folds). Carotene isomerase (*CRTISO*) gene was transcriptionally induced in both leaves and fruits of the nSe-treated seedlings by an average of 5.5 folds. Both BSe or nSe at the higher concentration increased proline concentrations, H_2_O_2_ accumulation, and lipid peroxidation levels, suggesting oxidative stress and impaired membrane integrity. Both BSe or nSe treatments also led to the induction of enzymatic antioxidants (catalase and peroxidase), an increase in concentrations of ascorbate, non-protein thiols, and soluble phenols, as well as a rise in the activity of phenylalanine ammonia-lyase enzyme. Supplementation at 3 mgl^-1^ improved the concentration of mineral nutrients (Mg, Fe, and Zn) in fruits. The bioaccumulated Se contents in the nSe-treated plants were much higher than the corresponding concentration of selenate, implying a higher efficacy of the nanoform towards biofortification programs. Se at 10 mgl^-1^, especially in selenate form, reduced both size and density of pollen grains, indicating its potential toxicity at the higher doses. This study provides novel molecular and physiological insights into the nSe efficacy for improving plant productivity, fruit quality, and fruit post-harvest longevity.

## Introduction

Nowadays, diverse attempts have been made to improve productivity, stress tolerance, and disease management in crops as well as to produce biofortified seeds or fruits containing minerals essential for humans. Zinc (Zn), iron (Fe), and selenium (Se) are the most important mineral nutrients that are often considered in biofortification programs. In this regard, the daily nutritional requirement of Se is estimated to be 55 micrograms. Taking nano-fertilizers and nano-pesticides into account, nanotechnology has provided a great opportunity to improve crop productivity and crop protection and has solved some of the challenges that we face today in agriculture. It has been highlighted in recent studies that bioavailability and function of Se in plant growth and metabolism were significantly more efficient in form of nanoparticles (nSe) compared to other natural Se forms such as selenate and selenite [1–6]. On the other hand, there is evidence that nSe use could potentially compromise plant growth and development [3, 5, 6]. However, the potential benefit or phytotoxicity of nSe is still controversial. Therefore, more detailed investigations are necessary to fill the knowledge gaps.

In plants, microRNAs (miRNAs) are transcriptionally modulated by endogenous and surrounding environmental cues [7]. Most miRNAs are efficiently involved in adjusting the perception/signaling of phytohormones, as well as regulating plant growth and development through transcriptional and posttranscriptional regulations of a multitude of target genes [8]. Some miRNAs play a significant role in adapting plants to different types of environmental stresses [7, 8]. Moreover, the flowering phase and fruit development are rigorously controlled through orchestrated molecular strategies in which miRNAs play critical roles at transcriptional and post-transcriptional levels [7]. In this regard, microRNA172 (miR172) is one of the most important miRNAs playing critical roles in controlling developmental programs, particularly at the flowering phase, as well as in conferring stress tolerance [8, 9]. To the best of our best knowledge, there has been no study investigating the role of miRNAs during plant responses to nanoparticles. In this study, we attempted to monitor transcriptional rates of miR172 following the nSe applications.

Carotenoid pigments are natural organic substances derived from an isoprene precursor. These pigments are involved in plant protection against oxidative stress and the photoinhibition process. Furthermore, these antioxidant metabolites provide numerous advantages to the health maintenance of humans owing to their great antioxidant characteristics. Therefore, investigating the effects of environmental cues, chemicals, and fertilizers on the concentrations of carotenoids is of great importance. In carotenoid production, the carotene isomerase (*CRTISO*) is one of the most important genes involved in the biosynthesis of carotenoids through mediating the conversion of ζ carotene to trans-lycopene [10–12]. *CRTISO* is widely considered in plant biotechnology programs to improve fruit quality and plant resistance to stresses [10–12]. Hence, the concentration of carotenoids in tomato fruit is considered an important aspect of fruit quality. Therefore, we aimed to highlight the potential alterations in fruit production, transcription of the *CRTISO* gene, and fruit longevity following foliar applications of nSe or selenate as a bulk (BSe).

The basic leucine zipper (bZIP) transcription factors are involved in a plethora of plant biological processes and responses, including signal transduction [6, 13], tissue differentiation [13], hormone signaling [14], nutrition [15], and stress tolerance [6, 16]. For this reason, evaluating the potential nSe-associated alteration in the bZIP transcription factor was also one of the aims of this study.

Within this framework, this study was carried out to address the possible responses of tomato seedlings to foliar application of BSe or nSe at different concentrations. In this manuscript, for the first time, we aimed to address transcriptional responses of *miR172*, *CRTISO*, and *bZIP* genes to foliar application of bulk Se (selenate) or nano counterpart (nSe) at the same concentration. Moreover, this study provides anatomical evidence highlighting the potential risk of Se or nSe on the development of pollen grains for the first time. Taking knowledge gaps into account, we attempt to present comparative comprehensive data on the Se- or nSe-mediated changes in plant growth, physiology, metabolism, the transcriptional program of genes, crop productivity, fruit quality, and fruit postharvest longevity to gain new insights into the benefits or the risk associated with Se function in agriculture. The main aims of this experiment are documenting the nSe-associated changes in (I) plant growth and flowering time (II) fruit production, quality, and postharvest life, (III) development of pollens in stamen, (IV) transcriptional responses of *miR172*, *CRTISO*, and *bZIP* genes, (V) enzymatic (catalase and peroxidase) and non-enzymatic (ascorbate and thiols) antioxidants, (VI) H_2_O_2_ concentration and membrane stability, (VII) proline concentration (a multifunctional protective amino acid), and (VIII) nutritional status in fruits.

## Material and methods

### Experimental conditions

Red elemental nSe (1000 mgl^−1^ containing polyvinylpyrrolidone (PVP) as a stabilizer; CAS#7446-08-4; APS:10–40 nm; true density: 3.89 gcm^−3^; morphology: near-spherical) was supplied from NanoSany Corporation, Mashhad, Iran. A sodium selenate (Na_2_SeO_4_) was supplied from TitraChem, Tehran, Iran.

In soilless conditions, seeds were grown in pots containing perlite and vermiculite (3:1) and irrigated with a Hoagland nutrient solution. The 37-day-old seedlings in the treatment groups were sprayed with different doses of nSe (0, 3, and 10 mgl^-1^) dissolved in deionized water and those in the control groups with different doses of sodium selenate (bulk Se (BSe); 0, 3, and 10 mgl^-1^). Seedlings were treated six times within 42 days, with one-week intervals between treatments. Growth-related traits (shoot fresh mass and root fresh mass), flowering time, number of fruits, and fruit postharvest longevity were recorded. Furthermore, molecular and physiological responses were monitored in leaves and fruits.

### Transcriptions of miR172, CRTISO, and bZIP genes

Total RNA was extracted using an RNA extraction kit (Promega-GRE#AS1500) from both leaves and fruits. The forward and reverse sequences (5'-3') of primers for miR172, bZIP, CRTISO, and GAPDH genes are displayed in Table 1. Quantitative polymerase chain reaction (qPCR) was performed using mi-1STEP RT-qPCR kit (Metabione, Co-GRE#mi-E8105), the transcription level of target genes were quantified using a PCR instrument (QIAGEN's real-time PCR cycler, Rotor-Gene Q) under the program specified by the manufacturer (reverse transcription in 50°C for 10–15 min; initial denaturation in 95°C for 5 min; denaturation in 95°C for 15 sec; annealing and elongation in 60–65°C for 1 min). Transcription rate was calculated using the equation 2^-ΔΔCT^ in which ΔCT was calculated by subtracting the internal control CT value from the CT amount of each target gene.

**Table 1:** The forward and reverse primer sequences for amplification of miR172, bZIP, CRTISO, and GAPDH genes.

Primer name	Sequence (5'-3')	Tm
*miR172*-F	ACACAGTTGTTGTTTGCAAATGT	54.4
*miR172*-R	TCTGACTCTCACCGATAGT	54.4
*bZIP*-F	GGACTTGTCATGGACCACAAT	60
*bZIP*-R	GCAAGACATCGGCAGTCATA	59.78
*CRTISO*-F	TGGAAGCACTGCAGACCATA	59
*CRTISO*-R	AGTACACAACACACACCGCT	59.8
*GAPDH*-F	ACGAATGCCGAGCATAGGAG	57.1
*GAPDH*-R	CCACCACTCGTGTACTGCAA	57.4

### Hydrogen peroxide (H_2_O_2_) concentration and lipid peroxidation level

H_2_O_2_ concentrations in leaves of BSe/nSe-treated plants were measured according to the procedure described by Sheteiwy et al. [17]. Briefly, 0.1% trichloroacetic acid (TCA) was applied to prepare leaf extract. After that, the resulting extract was centrifuged and the supernatant was separated. The reaction mixture consisted of 1000μL KI (1 mM), 500μL of potassium phosphate buffer (10 mM, pH 7.0), and 500 μL supernatant. Next, the absorbance was spectrophotometrically recorded at 390 nm. H_2_O_2_ concentration was calculated according to the equation of a standard curve. Membrane integrity was also evaluated by quantifying Malondialdehyde (MDA) contents in leaves according to the protocol described by Salah et al. [18] with a small modification. Briefly, fresh leaf tissue was grounded in 5 ml of 0.25% thiobarbituric acid (TBA) in trichloroacetic acid (TCA) (0.25 g TBA was dissolved in 100 ml of 10% TCA). The resulting homogenate was then placed at 95° for 20 minutes. Next, the samples were quickly cooled in an ice bath and centrifuged. The supernatant absorption was spectrophotometrically measured at 600 and 532 nm. Calculation of MDA was performed using the extinction coefficient of 155 mM^-1^cm and expressed in micromol per gram fresh weight (μMg^-1^fw).

### Activities of peroxidase, catalase, and phenylalanine ammonia-lyase (PAL) enzymes

In order to extract enzymes, phosphate buffer (0.1 M; pH of 7.2) was applied to the liquid nitrogen-grounded leaves. Next, the homogenate was centrifuged at 4°C and the resulting supernatant was stored at −80°C until enzyme analysis. The nSe-mediated variations in defense-related enzymes, including peroxidase [19], catalase [18], and PAL [20] were quantified in leaves. The peroxidase reaction was triggered by the addition of 100 μl enzyme extract to the reaction medium containing 100 μl guaiacol, 100 μl H_2_O_2_, and 2700 μl phosphate buffer (25 mM, pH 7.1). To estimate enzyme activity, the absorbance difference was monitored at 470 nm. Finally, the peroxidase activity was expressed in terms of Unit enzyme per gram fresh weight (UnitEg^-1^fw). To determine catalase activity, the reaction mixture containing 200 μl H_2_O_2,_ 200 μL enzyme extract, and 2600 μl phosphate buffer (25 mM, pH 7.1) were prepared. In this assessment, a decrease in absorbance at 240 nm was recorded following the addition of H_2_O_2_. To quantify PAL activity in leaves, 200 μl enzyme extract was added to the reaction mixture containing 6 μM phenylalanine and Tris-HCl buffer (500 mM, pH8). After that, the reaction mixture was incubated at 37°C for 1 hour. Then, the PAL reaction was stopped by the addition of 50 μl HCl (5 N). Finally, the PAL activity was determined according to the cinnamate standard curve and calculated in terms of microgram cinnamate per hour per gram fresh weight (μgCin.h^−1^g^−1^fw).

### Characterization of Mg, Fe, Zn, and Se concentrations

Ash solution was prepared by thermal decomposition in a furnace (650°C) and subsequently dissolving in nitric acid and hydrochloric acid (2:1). Mg, Zn, and Fe contents were assessed using Atomic Absorption Spectroscopy (AAS; Varian, Spectr AA.200). Furthermore, Se concentration was measured using a fully automatic Flame/Graphite Furnace AASAA.200; YOUNGLIN AAS 8020).

### Ascorbate, non-protein thiols, proline, and soluble phenols

The concentrations of non-protein thiols were quantified in leaves according to the protocol described by Del Longo et al. [21]. The leaves were homogenized in a 5% (w/v) solution of sulfosalicylic acid. The assay reaction of non-protein thiols consisted of 500μl DTNB (1 mM), 500μl phosphate buffer (100 mM, pH 7.1), 100μl of leaf extract, and 500 μM EDTA. After incubation for 10 minutes, the absorbance amount was recorded at 412 nm. The ascorbate contents were measured in leaves according to the protocol described by De Pinto et al. [22]. Briefly, metaphosphoric acid (5%) was used to prepare the leaf extract. Then, the resulting extract was centrifuged and the supernatant was separated. The reaction mixture was a 100 μl leaf extract, 250 μl phosphate buffer (50 mM, pH 7.2) supplemented with 50μl dithiothreitol (DTT; 10 mM), and 5 mM EDTA. After that, this mixture was incubated at room temperature for 10 minutes. After incubation for 10 min at room temperature, the process was followed by the addition of 150 μl N-ethylmaleimide (0.5%). Next, 200 μl dipyridyl of 4% in 70% ethanol, 200 μl orthophosphoric acid (44%), 200μl trichloroacetic acid (TCA; 10%), and 0.3% (w/v) FeCI_3_ were added and the mixture was vortexed. After incubation at 40°C for 40 minutes, the absorbance of each sample was recorded at 525 nm. Ascorbate concentration was calculated based on the standard curve equation of ascorbic acid. The concentration of proline amino acid in leaves was determined following the protocol described by Bates et al., [23]. To prepare leaf extract for proline assay, sulfa salicylic acid (3% w/v) was utilized. The ninhydrin reagent was prepared by mixing 30 ml acetic acid and 20 ml phosphoric acid (6 M). Next, 2 ml leaf extract was added to the reaction mixture containing 2 ml ninhydrin reagent and 2ml acetic acid (glacial). This mixture was heated at a boiling water bath for one hour. Then, this reaction mixture was immediately cooled down at an ice bath. After that, the reaction was followed by the addition of toluene solvent and vigorously shaking using a vortex. Then, the absorbance of the toluene phase was spectrophotometrically recorded at 520 nm. Proline concentration was estimated by using the equation of the standard curve of proline amino acid. Finally, Folin-Ciocalteu reagent was utilized to measure soluble phenols in leaves [24]. Briefly, total soluble phenols were extracted by homogenizing leaves in an ethanol solvent of 80% (v/v) and incubating in a boiling water bath. The reaction was started by the addition of 1 ml leaf ethanolic extract to a mixture containing 1 ml Folin-Ciocalteu reagent and saturated Sodium carbonate (21%). After 10 minutes and the development of a blue color, the mixture was centrifuged. The absorbance of the resulting supernatant was read at 760 nm. The concentration of total soluble phenols was quantified based on the equation of the standard curve of tannic acid.

### Histological study

The flower buds at the same developmental age were fixed in FAA fixator [6]. Longitudinal sections were made by a microtome and were stained using Hematoxylin/Eosin staining protocol. Stained sections were observed at different magnification levels and photographed using a light microscope [6].

### Statistical analysis

The experimental design was completely randomized. Every experiment was independently performed in the replication of three. All data were subjected to analysis of variance (ANOVA) using GraphPad software. The mean values of the three independent replications were compared using Tukey’s range test.

## Results

### Growth, biomass, productivity, and fruit postharvest longevity

**Fig 1:**
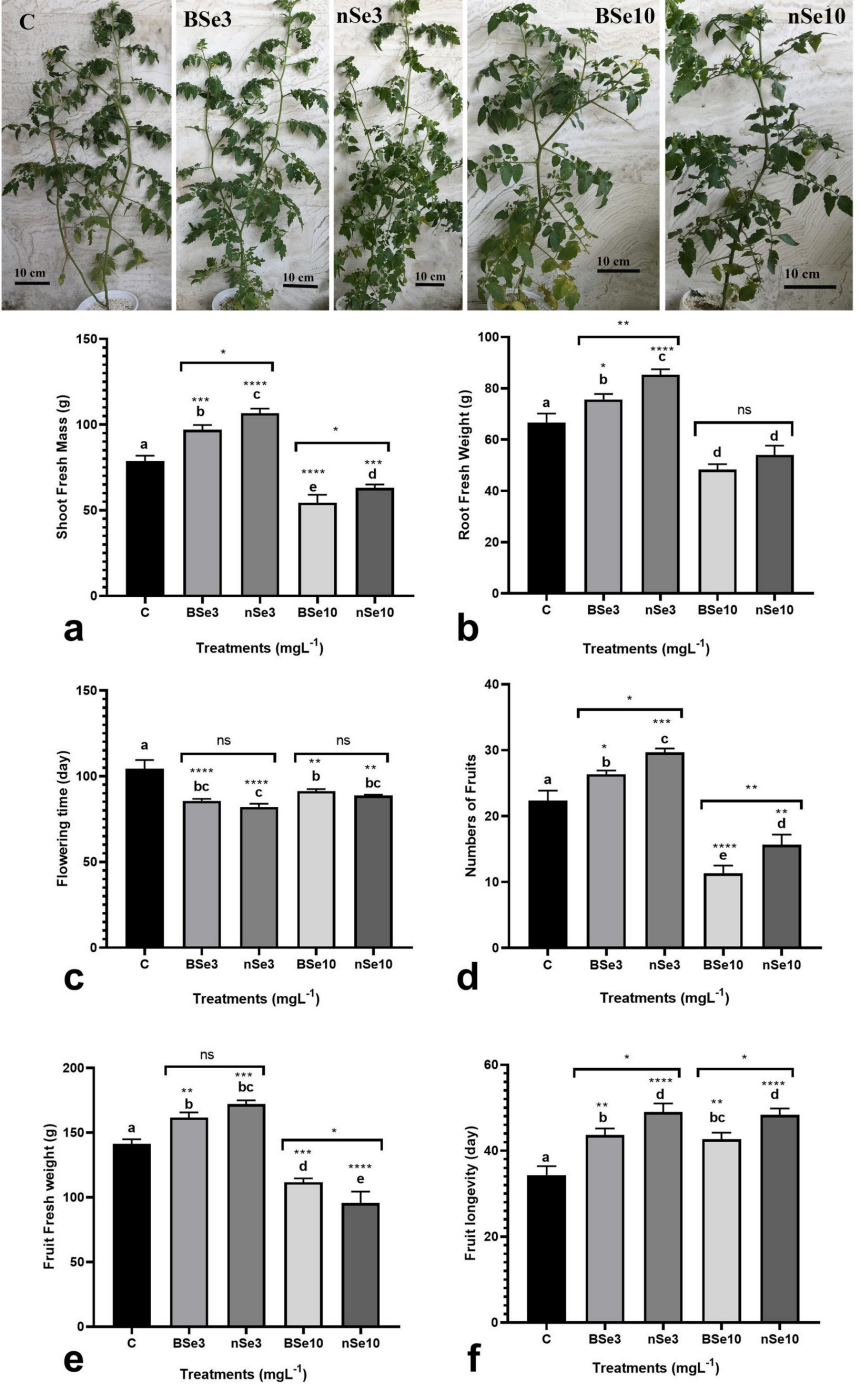
Variations in shoot and root biomass (a, b), flowering time (c), numbers of produced fruits (d), fruit biomass (e), and fruit postharvest longevity (f) in response to foliar application of BSe or nSe. Data are Mean ± standard deviation (SD) of three independent replications (n = 3). Treatment groups: C- Control; BSe3- Bulk Se at 3 mgl^-1^; nSe3- nSe at 3 mgl^-1^; BSe10- Bulk Se at 10 mgl^-1^; nSe10- nSe at 10 mgl^-1^; Different letters on top of columns refer statistically significant difference according to the Tukey's multiple comparisons test. ns: non-significant; *: 0.01<p≤0.05; **: 0.001<p≤0.01; ***: 0.0001<p≤0.001; ****: p≤0.0001. The asterisk on each column indicates a p-value level for comparing the mean of each group with the control group. Additionally, a comparison between the two Se groups at the same concentration is displayed by placing an asterisk (*) on the line.

To understand whether the nSe effectiveness in inducing growth, flowering, and yield changes are different from selenate, several traits, including shoot biomass, yield, fruit quality, and fruit postharvest life were evaluated. Compared with controls, both BSe and nSe treatments at 3 mgl^-1^ significantly increased fresh biomass in the shoot by 23% and 35%, respectively (Fig 1A). While BSe and nSe treatments at 10 mgl^-1^ led to a significant decrease in fresh biomass in the shoot, 31% and 20%, respectively, when compared with controls (Fig 1A). Similarly, treatment of seedlings with both BSe or nSe at 3 mgl^-1^ significantly improved fresh biomass in roots, by an average of 20.75%, whereas treatments at 10 mgl^-1^ adversely influenced root biomass by an average of 23% relative to the control (Fig 1B). However, all applied treatments accelerated the vegetative/reproductive stage switch and reduced flowering time (Fig 1C). The numbers of produced fruits were increased in response to foliar application of either BSe or nSe (especially the latter) at 3 mgl^-1^ by an average of 25.3% compared to the control (Fig 1D). On the other hand, the BSe or nSe treatment at 10 mgl^-1^ was associated with toxicity and restricted fruit production by a mean of 39.5% when compared to the control (Fig 1D). The fruit fresh mass in the seedlings treated with BSe3 or nSe at 3 mgl^-1^ was also higher than the control by an average of 18% (Fig 1E). The supplements at the high dose (10 mgl^-1^) not only reduced fruit production but also significantly reduced fruit fresh weight (Fig 1E). Finally, treatments at all applied concentrations, significantly improved fruit postharvest longevity by an average of 38% (Fig 1F).

### Transcriptional responses of genes *miR172*, *bZIP* transcription factor, and *CRTISO*

**Fig 2:**
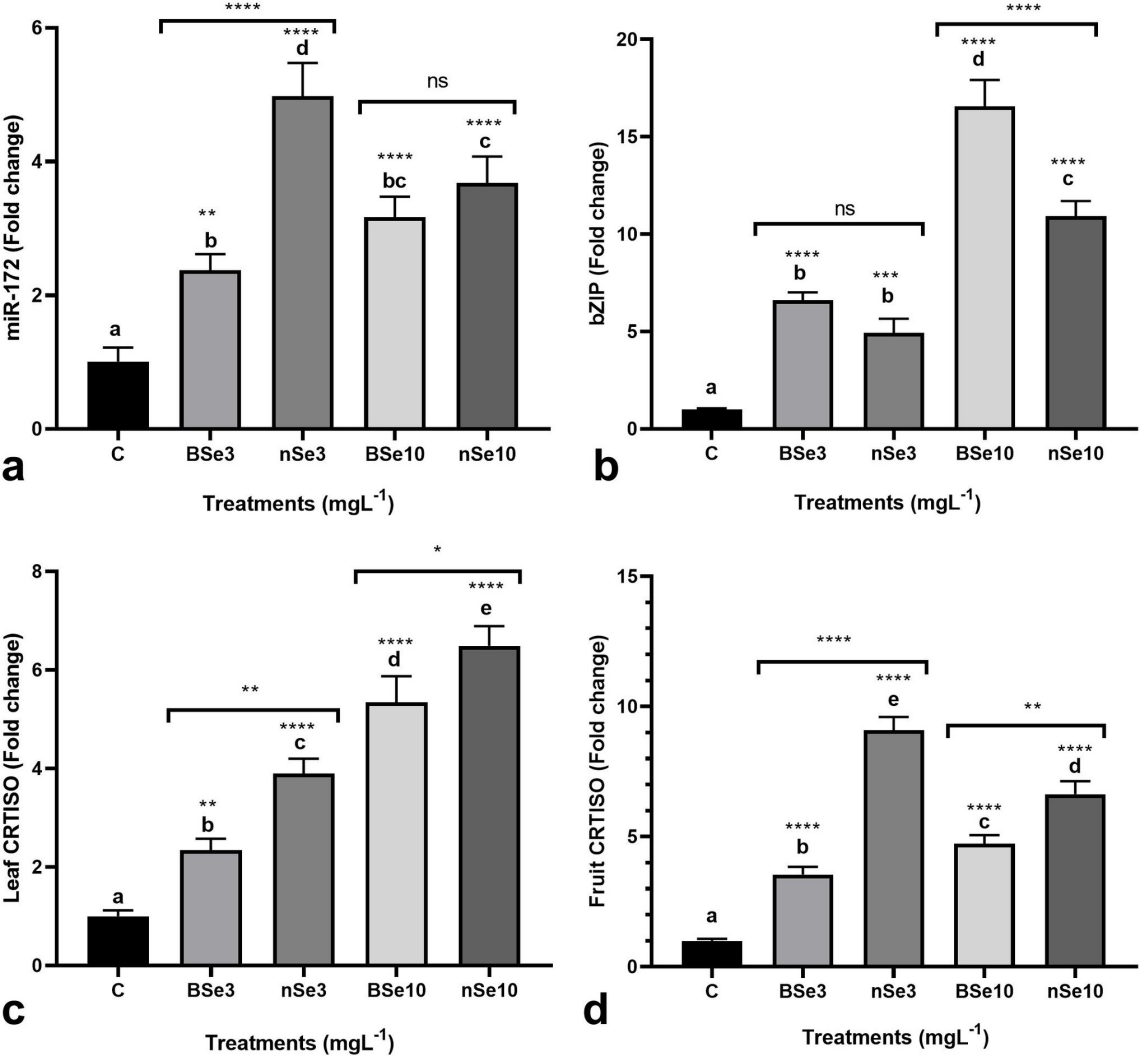
BSe or nSe-mediated transcriptional changes in leaf *miR172* (a), leaf *bZIP* transcription factor (b), leaf *CRTISO* (c), and fruit *CRTISO* (d); Data are Mean ± standard deviation (SD) of three independent replications (n = 3). Treatment groups: C- Control; BSe3- Bulk Se at 3 mgl^-1^; nSe3- nSe at 3 mgl^-1^; BSe10- Bulk Se at 10 mgl^-1^; nSe10- nSe at 10 mgl^-1^; Different letters on top of columns refer statistically significant difference according to the Tukey's multiple comparisons test. ns: non-significant; *: 0.01<p≤0.05; **: 0.001<p≤0.01; ***: 0.0001<p≤0.001; ****: p≤0.0001. The asterisk on each column indicates a p-value level for comparing the mean of each group with the control group. Additionally, a comparison between the two Se groups at the same concentration is displayed by placing an asterisk (*) on the line.

In addition to the growth-related responses, BSe or nSe-mediated changes in the expression of target genes were investigated to provide comparative molecular evidence, for the first time. In leaves, the BSe3, nSe3, BSe10, and nSe10 treatments transcriptionally upregulated miR172 by 2.4, 4.6, 3.2, and 3.9 folds, respectively (Fig 2A). likewise, the BSe or nSe treatments at the lower concentration (3 mgl^-1^) moderately stimulated the expression of the *bZIP* gene (mean = 5.7 folds) (Fig 2B); while, the supplements at the higher dose (10 mgl^-1^), mediated drastic transcriptional upregulation in the *bZIP* gene (mean = 13.7 folds) (Fig 2B). Similarly, the transcription of the *CRTISO* gene was upregulated in leaves of the seedlings treated with BSe or nSe (mean = 4.5 folds); the highest expression was observed in seedlings treated with BSe or nSe at 10 mgl^-1^ (Fig 2C). In fruit, the *CRTISO* gene was also found to be transcriptionally upregulated in response to the supplements by an average of 5.9 folds (Fig 2D).

### Physiological responses

**Fig 3:**
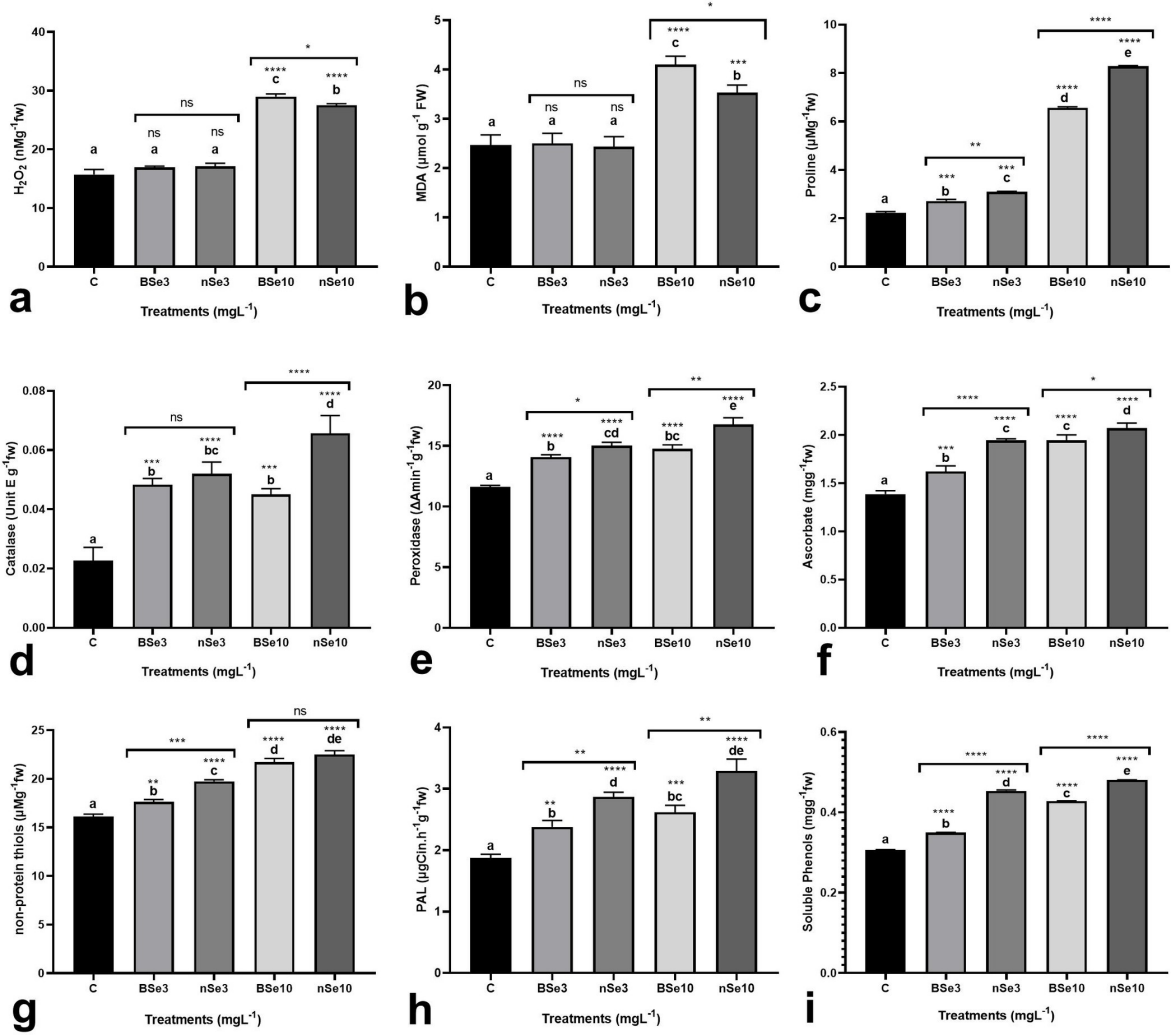
Variations in different physiological traits in response to foliar application of BSe or nSe. Data are Mean ± standard deviation (SD) of three independent replications (n = 3). Treatment groups: C- Control; BSe3- Bulk Se at 3 mgl^-1^; nSe3- nSe at 3 mgl^-1^; BSe10- Bulk Se at 10 mgl^-1^; nSe10- nSe at 10 mgl^-1^; Different letters on top of columns refer statistically significant difference according to the Tukey's multiple comparisons test. ns: non-significant; *: 0.01<p≤0.05; **: 0.001<p≤0.01; ***: 0.0001<p≤0.001; ****: p≤0.0001. The asterisk on each column indicates a p-value level for comparing the mean of each group with the control group. Additionally, a comparison between the two Se groups at the same concentration is displayed by placing an asterisk (*) on the line.

To evaluate the occurrence of oxidative stress, activation of the defense system, and changes in metabolism in response to BSe or nSe utilization, several physiological traits were measured. In this regard, H_2_O_2_ concentration and MDA content (membrane integrity index) were monitored to estimate the BSe- or nSe-associated risk of oxidative stress. To monitor the activation of the defense system, the BSe- or nSe-mediated changes in enzymatic antioxidants (catalase and peroxidase), non-enzymatic antioxidants (ascorbate and non-protein thiols), and proline concentration were assayed. PAL activity and concentration of total soluble phenols were measured to address the BSe- or nSe-associated alteration in secondary metabolism. The BSe or nSe treatments at 10 mgl^-1^ led to a drastic rise, of about two folds, in the H_2_O_2_ contents of leaves (Fig 3A). The BSe or nSe treatments at 3 mgl^-1^ did not make a statistically significant change in membrane integrity (Fig 3B) but made a slight increase in leaf proline concentration (Fig 3C). Additionally, the BSe or nSe treatments at a high dose (10 mgl^-1^) drastically increased lipid peroxidation level as well as in the leaf proline content (Fig 3B and 3C). Catalase enzyme activity was significantly higher in all seedlings treated with BSe or nSe, by a mean of 2.2 folds when compared to the controls at all applied concentrations (Fig 3D); similarly, peroxidase activity and ascorbate concentration were higher in leaves, by an average of 30% and 36%, respectively (Fig 3E and 3F). With increasing the BSe or nSe concentration, the non-protein thiols were also increased linearly by an average of 27% (Fig 3G). Furthermore, treatments with the BSe3 (26.8%), nSe3 (52%), BSe10 (39.6%), and nSe10 (75.3%) treatments significantly stimulated the PAL activity in leaves (Fig 3H). Likewise, Se treatment, particularly the nSe treatment, significantly increased concentrations of the soluble phenols by an average of 39% (Fig 3I).

### Nutritional status in fruit

**Fig 4:**
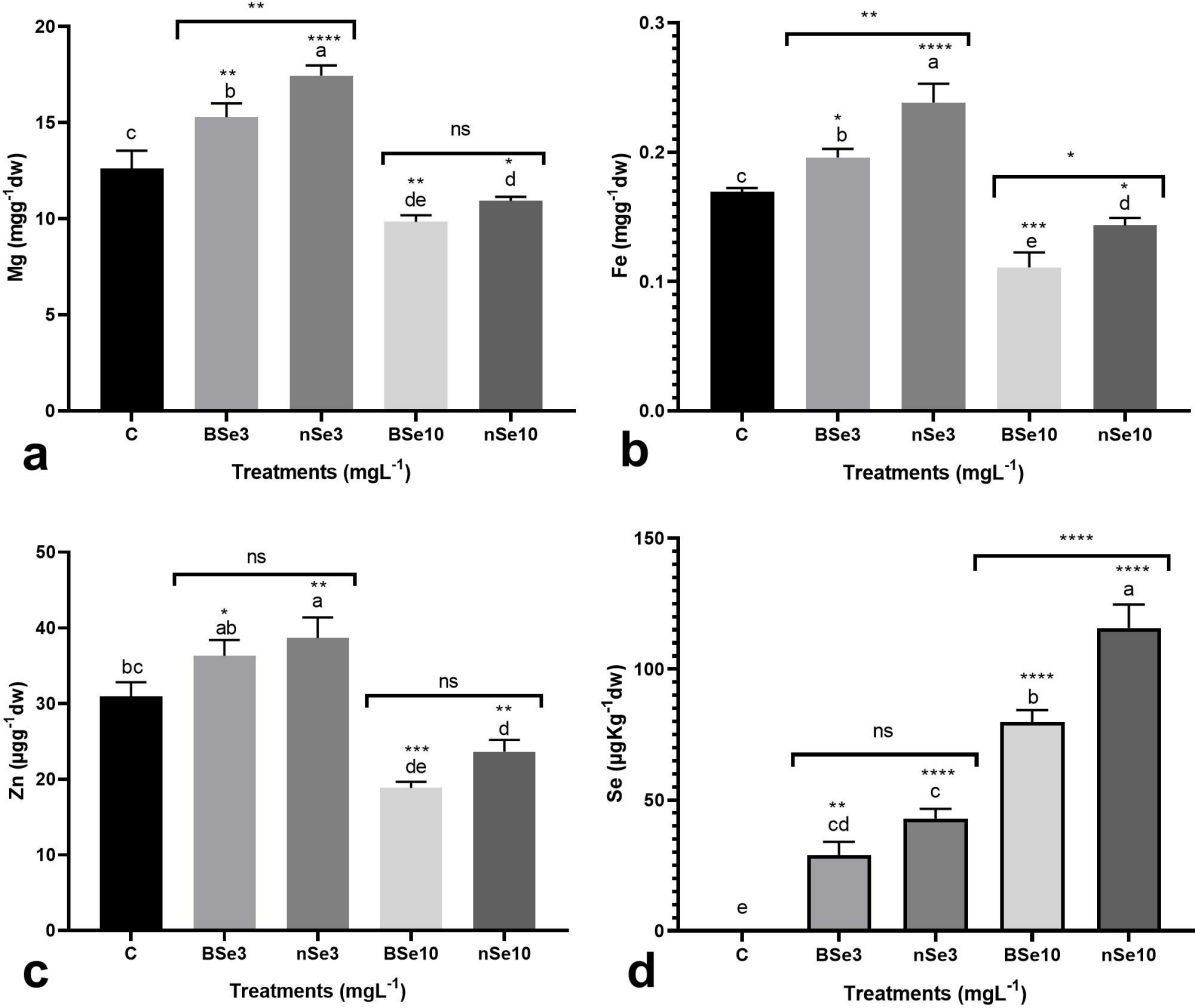
BSe/nSe-mediated changes in Mg (a), Fe (b), Zn (c), and Se (d) concentrations in fruits. Data are Mean ± standard deviation (SD) of three independent replications (n = 3). Treatment groups: C- Control; BSe3- Bulk Se at 3 mgl^-1^; nSe3- nSe at 3 mgl^-1^; BSe10- Bulk Se at 10 mgl^-1^; nSe10- nSe at 10 mgl^-1^; Different letters on top of columns refer statistically significant difference according to the Tukey's multiple comparisons test. ns: non-significant; *: 0.01<p≤0.05; **: 0.001<p≤0.01; ***: 0.0001<p≤0.001; ****: p≤0.0001. The asterisk on each column indicates a p-value level for comparing the mean of each individual group with the control group. Additionally, a comparison between the two Se groups at the same concentration is displayed by placing an asterisk (*) on the line.

Fruit quality is an important determining factor in terms of fruit longevity and human nutrition. In this regard, the BSe or nSe-mediated changes in concentration of several important minerals were monitored. The Se supplementation altered concentrations of minerals in fruits in a dose-dependent manner (Fig 4A–4C). At 3 mgl^-1^, the BSe or nSe, especially the latter, significantly increased Mg, Fe, and Zn concentrations in fruits by averages of 29.8%, 27.6%, and 21%, respectively, when compared to the control (Fig 4A–4C); while supplementation at high dose (10 mgl^-1^) moderately decreased Mg, Fe, and Zn contents in comparison to the control (Fig 4A–4C). Furthermore, the foliar applications of both BSe or nSe led to Se-bioaccumulation in tomato fruits (Fig 4D); with nSe treatments leading to a higher amount of bioaccumulated Se in fruits compared with the bulk treatments (Fig 4D).

### The density of pollen grains in the pollen sac

**Fig 5:**
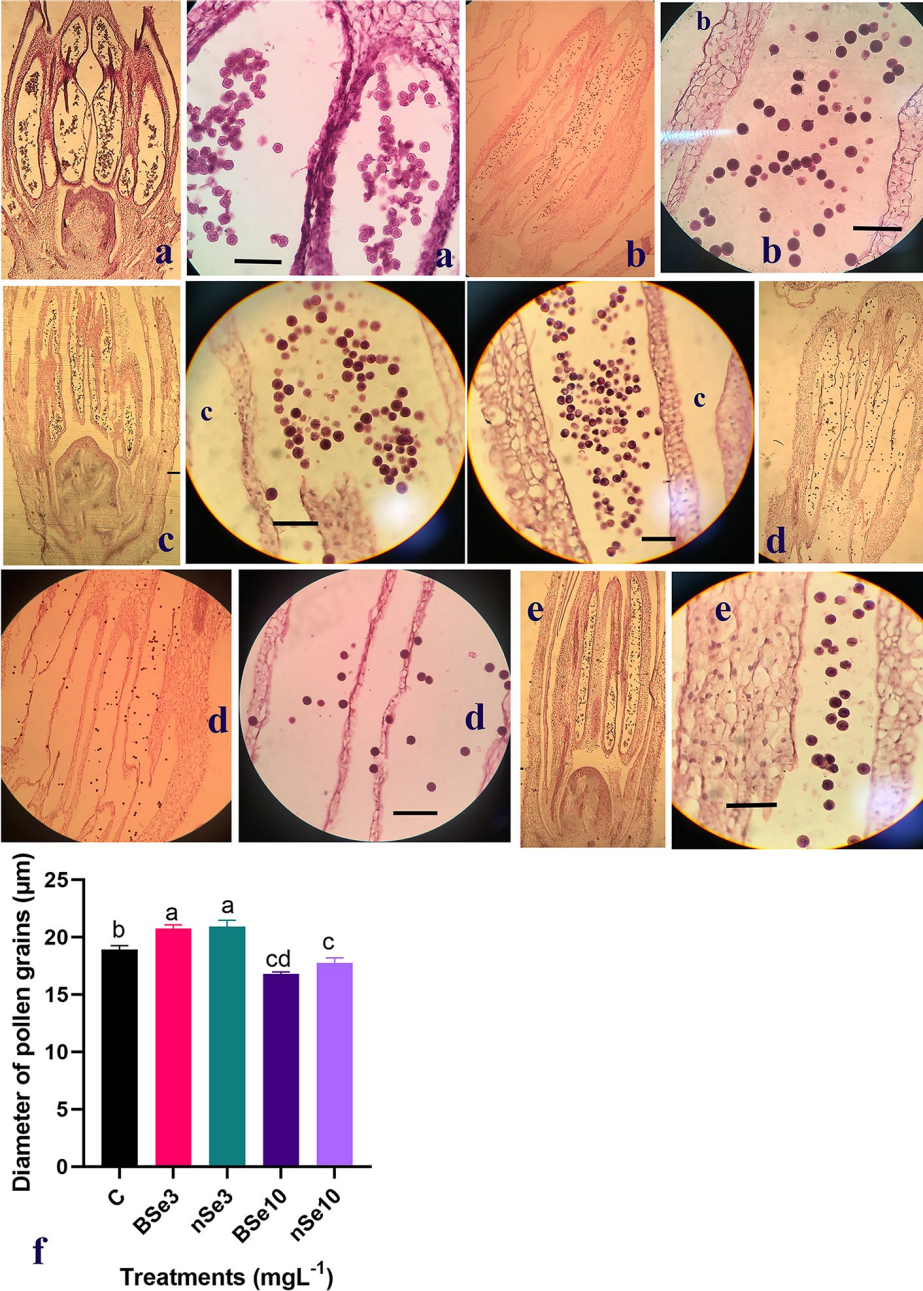
Longitudinal-sections of pollen sac of the BSe/nSe-treated plants, showing differential sizes and density of pollen grains. The drawn lines in each section indicate 100 μm. a- Control; b- BSe at 3 mgl^-1^; c- nSe at 3 mgl^-1^; d- BSe at 10 mgl^-1^; e- nSe at 10 mgl^-1^; f- mean diameter of pollen grains.

As mentioned above, our results showed that a high concentration of BSe or nSe significantly reduced crop yield. Hence, the BSe- or nSe-associated toxicity on the pollen grain development was evaluated for the first time. Assessment of the longitudinal sections of the flower buds indicated that the size and density of pollen grains were affected by the BSe/nSe treatments in a dose- and material type-dependent manners (Fig 5A–5F). The BSe or nSe treatments at all concentrations were associated with a decreased density of pollen grains in pollen sacs. However, the size of pollen grains increased with supplementation at 3 mgl^-1^. It is important to note that Se treatment at 10 mgl^-1^, especially in form of BSe, reduced both the size and the density of pollen grains, highlighting its potential toxicity at the higher dose (Fig 5D–5F).

## Discussion

According to our findings, the potential benefits of nSe utilization toward improving plant growth, biomass accumulation, yield, nutrition, flowering time, fruit quality, and postharvest longevity were much higher than the use of selenate, and at the same time, the potential nSe-associated risks are fewer. These results are consistent with several recent reports [5, 6, 25, 26]. The following mechanisms appear to mediate partly differential responses to nSe relative to the selenate; (I) variations in uptake kinetics (nSe inactive influx through aquaporins vs secondary active influx mechanism of selenate through symporter) [2], (II) differences in their interactions with biomolecules and metabolism into the organic forms [2, 26], (III) differential physicochemical properties of nanoparticles by which a nSe interaction with biomolecules can be different from that of selenate [2, 5, 6, 26], (IV) variation in a nSe-mediated change in phytohormones [3, 4, 27], and (V) epigenetic modification [5, 6]. However, the involved mechanisms are still poorly characterized which further emphasizes the need for further studies.

This study provides the first comparative evidence on the Se- or nSe-mediated variations in miRNAs as key regulatory checkpoints. The observed selenate/nSe-associated changes in developmental vegetative/reproductive switch, fruit-related characteristics, and plant physiology may be attributed to the *miR172* involvements. However, future transcriptome and proteome studies can improve our knowledge, fill knowledge gaps, and validate or disprove this hypothesis. Earlier reports suggested this hypothesis that miR172 acts a critical role in modulating plant reproduction, developmental programs, and conferring stress tolerance [9, 28, 29]. Moreover, this experiment provides an important molecular mechanism on how Se or nSe application can improve photosynthesis performance, confer stress tolerance, and increase fruit quality through a transcriptional up-regulation in *CRTISO*. Carotenoid concentration closely correlates with the expression of the *CRTISO* gene [10–12], and with conferring stress tolerance [12]. Several studies showed cytoprotective effects of Se [30–33] and nSe [1, 25, 27] at low doses during stress conditions in plants. We concluded that the *bZIP* transcription factor is more responsive to selenate whereas *CRTISO* and *miR*-172 are more responsive to the nSe supplementation. Recently, Sotoodehnia-Korani et al. [6] reported that the bZIP transcription factor is a responsive gene to nSe. However, they did not monitor the plant's response to selenate. The observed transcriptional responses can be explained by Se or nSe-associated variations in redox status [25], phytohormones [4, 27], and epigenetic modification [5, 6]. An almost similar trend in the expression of the target genes (*CRTISO*, *bZIP*, and *miR-172*) and the physiological traits suggests the involvement of similar and convergent signaling pathways in response to selenate and nSe. However, significant differences in terms of transcription rates in the nSe-treated plants relative to selenate can be a good sign of partly differential signal perception, transduction, and signaling cascades. Several recent studies have addressed transcriptional responses to nSe, namely, *HSFA4A* in wheat [34], *RAS* and *HPPR* in *Melissa officinalis* [3], a *bZIP* transcription factor in pepper [6], *DREB1A*, *PAL*, *HCT1*, and *HQT1* genes in chicory [25], and *WRKY1*, *PAL*, and *4CL* in bitter melon [5]. However, these studies have only reported a response to the nano form. This research can, therefore, provide a new perspective in terms of comparing molecular responses to selenate and nSe.

**Fig 6:**
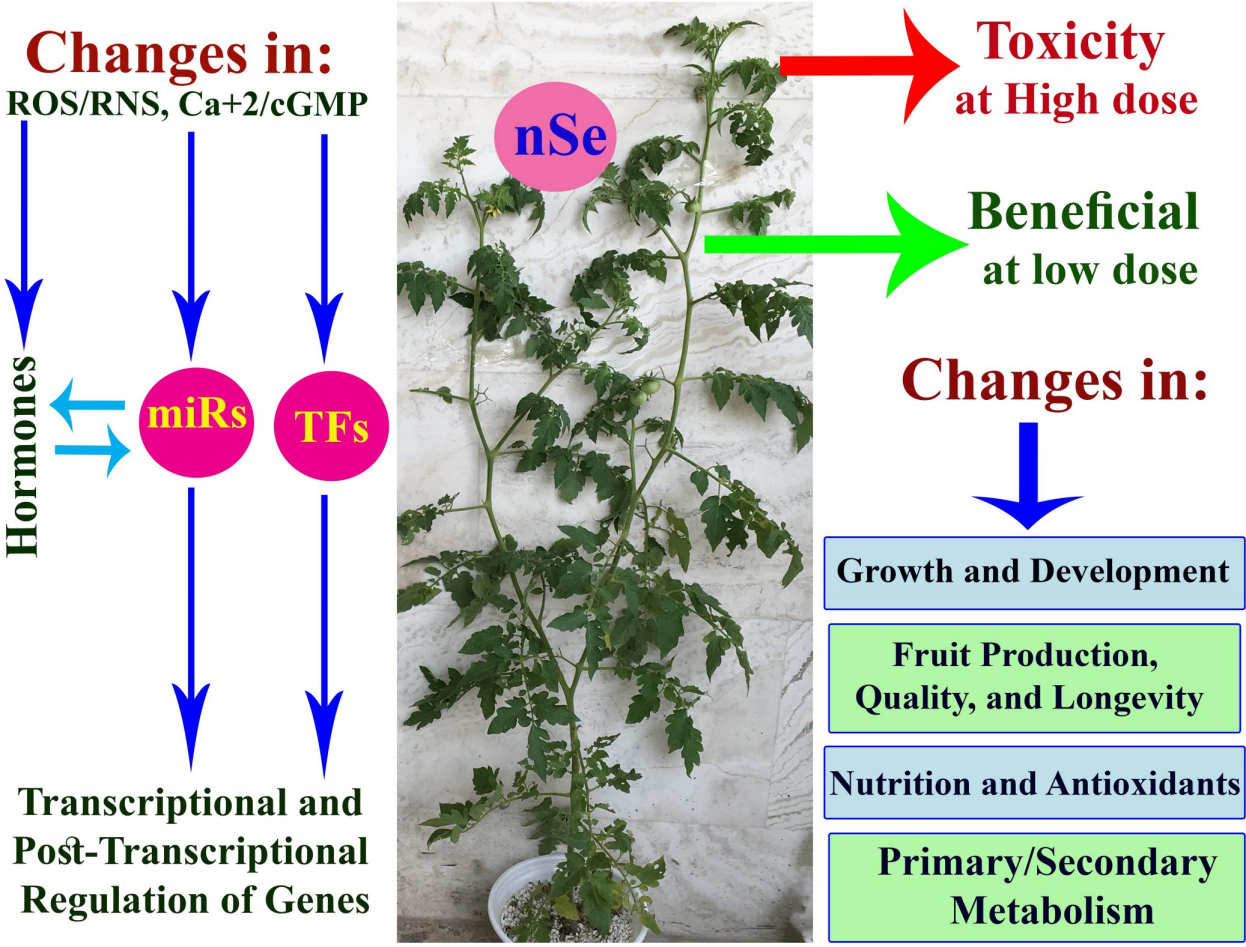
A schematic design on the potential molecular mechanisms contributed to plant responses to exogenously applied nSe. TFs: transcription factors; miRs; microRNAs; ROS; reactive oxygen species; RNS: reactive nitrogen species; cGMP: Cyclic guanosine monophosphate.

According to earlier literature, there is a knowledge gap in the field of potential changes in signaling molecules and redox-based regulation of cellular transcription program in response to Se, nSe, and other kinds of nanoparticles. In this study, we monitored H_2_O_2_ only once which is not good enough for addressing H_2_O_2_ fluctuation. Owing to the close relationship of H_2_O_2_ with other signaling molecules, the monitoring of potential fluctuations in nitric oxide, H_2_O_2_, and H_2_S is, therefore, recommended in designing future studies. Fig 6 displays schematics of the potential molecular mechanisms contributing to the plant responses to exogenously applied nSe.

This study provides the first comparative data in terms of physiological responses to selenate and nSe. According to the H_2_O_2_ accumulation and MDA level, Se or nSe at the low dose did not mediate oxidative stress. It seems that Se or nSe signaling at low concentration occurs through a slight transient increase in the H_2_O_2_ concentration which is subsequently detoxified by an increase in enzymatic antioxidants. H_2_O_2_ signaling triggers the transcriptional changes in downstream genes and activation of the defense system, including antioxidants and secondary metabolism. These physiological responses along with the observed molecular changes suggest mechanisms by which Se or nSe utilization, especially the nanoform, can improve crop tolerance to environmental stresses. However, it should be warned that these supplements at the high dose were associated with induction in the oxidative burst, impaired membrane integrity, disrupted metabolism, and growth disorders. The lower concentration of H_2_O_2_ and MDA along with higher content of proline in the nSe10-treated group is another reason for the lower toxicity of the nano form relative to the selenate counterpart. The nSe-mediated changes in proline, a multifunctional protective amino acid, have been attributed to the modified nitrogen assimilation metabolism [24], and activation of the defense system [5, 6]. Se or nSe supplementation, especially the latter, stimulated enzymatic antioxidants, non-enzymatic antioxidant compounds, and secondary metabolism. In agreement with our results, the nSe treatment improved enzymatic antioxidants [5, 6, 25], non-enzymatic antioxidants [25], and accumulation of secondary metabolites [3]. However, previous reports have only reported physiological responses to nSe. To the best of our knowledge, this study, for the first time, showed that nSe is more efficient than selenate in activating the antioxidant system and stimulating secondary metabolism.

The results confirmed a higher efficacy of the nano type for application in biofortification programs. Our results on Se-associated modification in nutritional status are in line with the findings of Amirabad et al., [33] in radish, Babajani et al. [3] in lemon balm, Alam et al. [32] in mung bean, and Zahedi et al. [4] in strawberry. Along with these reports, the nSe-associated modifications in vascular conducting tissues (xylem and phloem) [5, 6] may be responsible for the alterations in plant nutritional status. The higher fruit longevity, an economically important index, in the nSe-supplemented plants can be explained by a higher efficiency of nSe in enhancing the antioxidant system, improving minerals, and the antimicrobial properties of nSe when compared to that of selenate.

The results highlight how Se or nSe-mediated changes in vegetative growth and metabolism are coordinated with alteration in plant productivity at a reproductive stage. Herein, the provided anatomical evidence, for the first time, underlines the risk of Se at high concentration in restricting crop fertility through disrupting the development of pollen grains. It has been recently reported in pepper [6] and bitter melon [5] plants that in vitro toxicity of nSe is associated with impaired tissue differentiation and abnormalities in stem’s apical meristem. Further anatomical and developmental investigations of Phyto-toxicological concerns of nanomaterials are required to improve our knowledge of plant responses to nanoproducts, especially nano-pesticides and nano-fertilizers [5, 35–37].

## Conclusion

Taken together, this study highlights the considerable efficacy of Se or nSe, especially the nano form, for modifying growth, yield, primary and secondary metabolism, nutrition, defense system, and transcription program. The Se- or nSe-associated transcriptional changes in *miR172*, *CRTISO*, and *bZIP* are introduced as key potential molecular mechanisms through which Se application may confer stress tolerance. Moreover, this study provided anatomical evidence displaying potential phytotoxicity of Se or nSe in terms of disrupting the development of pollen grains. Taking knowledge gaps into account, these comparative comprehensive data can be helpful to gain novel insights into the benefits or the risk associated with Se or nSe function in agriculture. This study underlines the necessity of including anatomical, developmental, and molecular assessments in future phyto-toxicological investigations of nanomaterials.

### CRediT authorship contribution statement

Maryam Neysanian: Resources, methodology, review, and editing; Alireza Iranbakhsh: Conceptualization, visualization, investigation, formal analysis, writing-original draft, review, and editing; Rahim Ahmadvand: Conceptualization, visualization, investigation, software analysis, review, and editing; Zahra Oraghi Ardebili: Conceptualization, Investigation, review, and editing. Mostafa Ebadi: Conceptualization, visualization, investigation, formal analysis, writing, review, and editing. All authors have contributed, seen, and approved the manuscript.

## Supporting information

S1 Data (XLSX)
