# Correction: Comparative efficacy of selenate and selenium nanoparticles for improving growth, productivity, fruit quality, and postharvest longevity through modifying nutrition, metabolism, and gene expression in tomato; potential benefits and risk assessment

**DOI:** 10.1371/journal.pone.0250192

**Published:** 2021-04-08

**Authors:** Maryam Neysanian, Alireza Iranbakhsh, Rahim Ahmadvand, Zahra Oraghi Ardebili, Mostafa Ebadi

There is an error in affiliation 2 for author Rahim Ahmadvand. The correct affiliation 2 is: Department of Vegetables Research, Seed and Plant Improvement Institute, Agricultural Research, Education & Extension Organization, Karaj, Iran.
